# Supplementary material for: From Development to Regeneration: Insights into Flight Muscle Adaptations from Bat Muscle Cell Lines
Source: Cells. 2025 Aug 1;14(15):1190. doi: 10.3390/cells14151190 (PMC12346413; doi:10.3390/cells14151190)
Supplement: Supplementary file 1 [file cells-14-01190-s001.zip › cells-3764056-supplementary.pdf]

Table S1. A curated list of candidate genes included in the illustrated model of flight muscle at rest and following glucose stimulation (Figure 1), with functional annotations and known roles in skeletal muscle.

Gene	Functional Module	Relative to HK	DEG	Biological Role in Muscle
**Atp2a1**	Calcium cycling	> HK	No	Calcium reuptake in fast-twitch muscle
**Atp2a2**	Calcium cycling	> HK	No	Calcium handling in slow/oxidative muscle
**Casq1**	Calcium cycling	> HK	No	Calcium buffering in sarcoplasmic reticulum (fast muscle)
**Casq2**	Calcium cycling	> HK	No	Calcium buffering (cardiac/oxidative muscle)
**Cat**	Redox	> HK	No	ROS detoxification enzyme (catalase)
**Foxo3**	Redox	> HK	Yes	Redox regulation, muscle atrophy signaling
**Sod1**	Redox	> HK	No	ROS scavenging (superoxide dismutase)
**Txnip**	Redox	> HK	No	Redox regulation and glucose sensing
**Tmc5**	Muscle regeneration	< HK	Yes	Transmembrane channel; unclear function in muscle
**Mymx**	Muscle regeneration	> HK	Yes	Myoblast fusion
**Myh4**	Calcium cycling	> HK	Yes	Fast-twitch muscle fiber myosin
**Pak1**	Energy sensing	> HK	Yes	Energy sensing, insulin signaling
**Plk2**	Stress sensing	> HK	Yes	Stress-responsive kinase
**Chad**	Muscle regeneration	> HK	Yes	ECM remodeling in skeletal muscle
**Osbp2**	Lipid handling	> HK	Yes	Oxysterol binding, lipid sensing
**Csrp3**	Muscle regeneration	> HK	Yes	Z-disk protein, structural integrity
**Lingo4**	Muscle regeneration	> HK	Yes	Poorly characterized; possibly involved in regeneration
**Hey2**	Muscle regeneration	> HK	Yes	Myogenic transcription factor (Notch target)
**Pax7**	Muscle regeneration	> HK	Yes	Muscle stem cell maintenance
**Ucp2**	Redox	> HK	Yes	Mitochondrial uncoupling, redox regulation
**Tmeff1**	Muscle regeneration	> HK	Yes	Muscle regeneration and differentiation
**Map4k3**	Energy sensing	> HK	Yes	Nutrient signaling (mTOR, JNK)
**Nrf1**	Redox	> HK	Yes	Mitochondrial biogenesis and oxidative metabolism
**Slc4a7**	Energy sensing	> HK	Yes	Proton-coupled ion transporter (pH regulation)
**Maf**	Redox	> HK	Yes	Redox-responsive transcription factor
**Prkaa2**	Energy sensing	> HK	Yes	AMPK catalytic subunit (energy sensing)
**Etv5**	Stress sensing	< HK	Yes	Stress-responsive transcription factor
**Sdc3**	Muscle regeneration	< HK	Yes	Cell adhesion, muscle stem cell regulation
**Entpd3**	Stress sensing	< HK	Yes	Purinergic signaling; immune/stress response

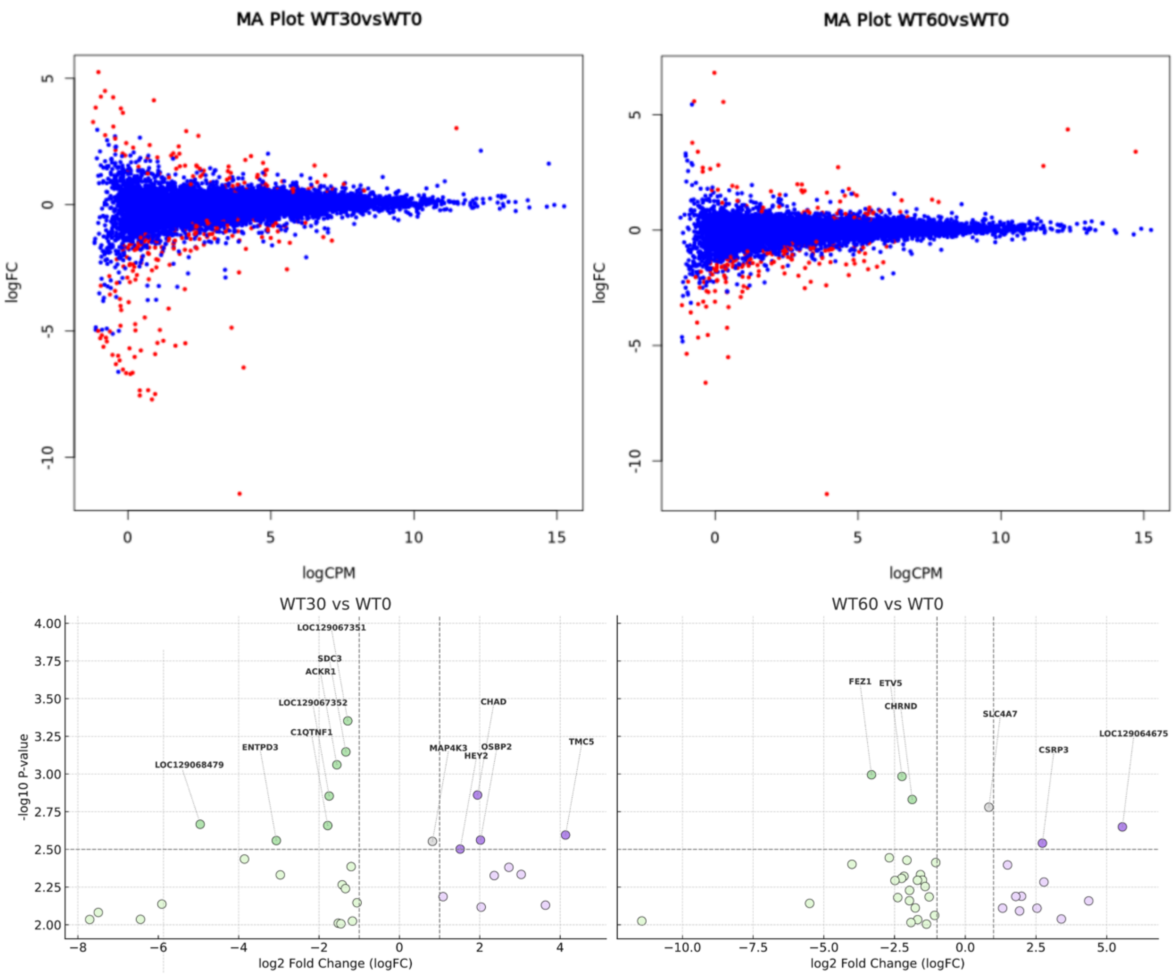

Figure S1: Differential gene expression in bat flight muscle following glucose stimulation. MA and volcano plots showing differential gene expression between 30 min post-glucose (WT30) and baseline (WT0) and between 60 min post-glucose (WT60) and baseline (WT0). Each point represents a gene. MA plots: All genes with a $\log_2$ fold change (logFC) versus average expression (logCPM) for differential gene expression in flight muscle following glucose stimulation. Red points indicate genes identified as significantly differentially expressed ($|\log_2 FC| > 1.0$, $p < 0.05$), while blue points indicate non-significant genes. These plots display the full dataset, allowing visualization of expression-dependent fold change patterns. Volcano plots: Most significantly differentially expressed genes. Darker violet (upregulated) and green (downregulated) indicate $-\log_{10}$(p-value) > 2.5 ($p < 0.0032$); lighter shades indicate the same fold change with lower statistical support ($-\log_{10}$(p-value) ≤ 2.5). Genes with $|\log_2$ fold change$| \leq 1$ are shown in gray. Dashed lines denote fold change thresholds (±1) and the significance cutoff ($p = 0.0032$). Genes passing both thresholds are labeled. These include transcription factors, signaling molecules, and membrane-associated proteins hypothesized to contribute to metabolic remodeling and stress responses in flight muscle. Left: Upregulated genes include *Chad*, *Sdc3*, *Heye2*, *Map4k3*, *Osbp2*, and *Tmc5*, associated with membrane remodeling, regeneration, and intracellular signaling. Downregulated genes include *LOC129067351* and *LOC129067352*, which show homology to MHC class II histocompatibility antigens (DR and DQ chains), suggesting transient suppression of immune activation. *Etv5*, a stress-responsive transcription factor, also decreases. Right: Upregulated genes include *Slc4a7* and *Csrp3*, linked to ion homeostasis and cytoskeletal organization. *LOC124900675*, a strongly upregulated uncharacterized non-coding RNA, is noted as a candidate regulatory transcript.

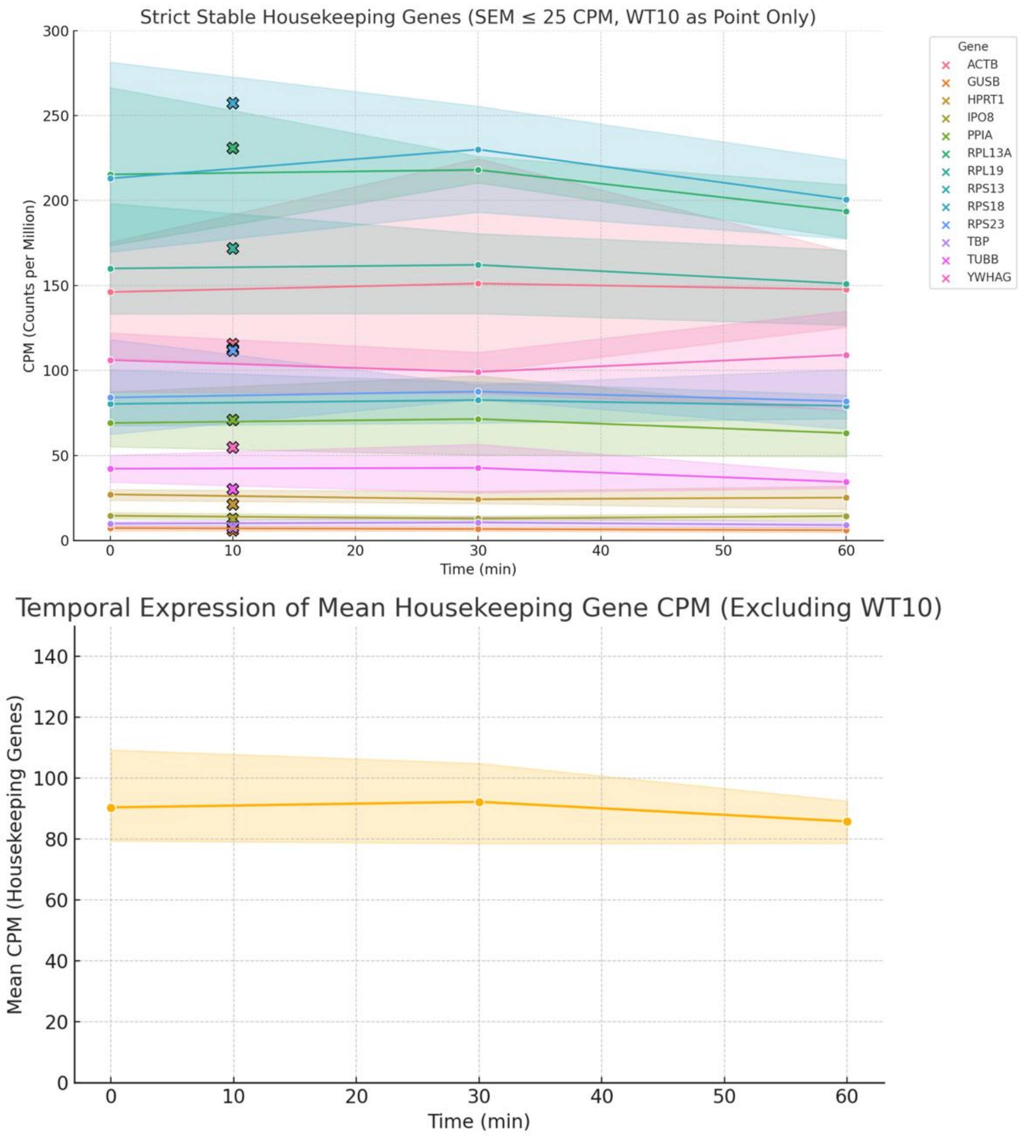

Figure S2. Housekeeping genes are stable across conditions. (Top) Counts per million (CPM) for individual housekeeping genes (n = 13) across timepoints 0, 30, and 60 min post-stimulation. Genes were selected based on expression stability (SEM ≤ 25 CPM) and average expression ≥ 25 CPM. Shaded regions represent SEM. WT10 values are shown as points only and were excluded from model fitting due to limited replication. **(Bottom)** Mean CPM for all strict housekeeping genes (excluding WT10), showing stable expression across time. Shading denotes SEM. These genes serve as a reference for identifying biologically meaningful expression above baseline transcriptional activity.

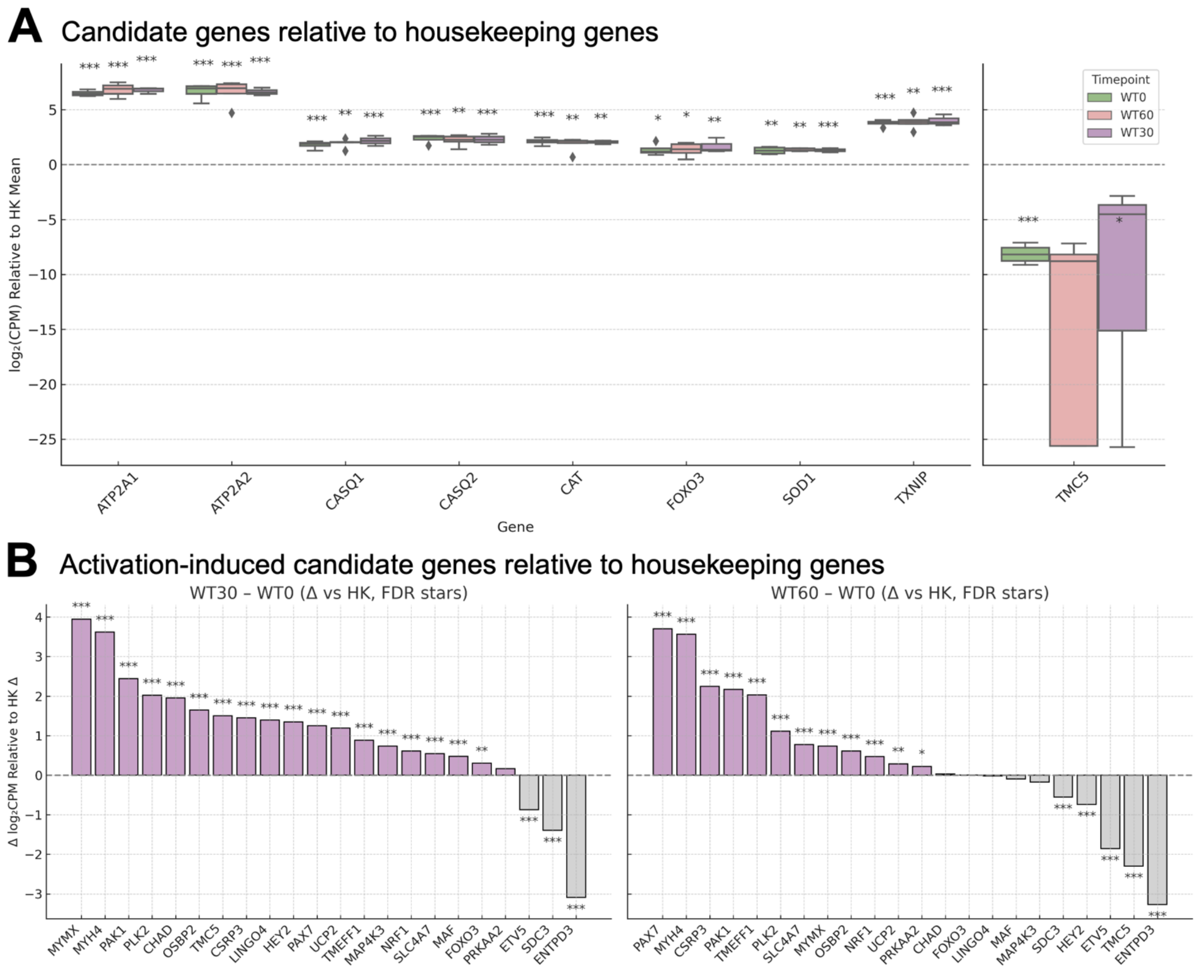

Figure S3: Gene expression changes relative to housekeeping genes. A. Boxplots show $\log_2$-transformed CPM (counts per million) values of candidate genes involved in redox buffering (*Foxo3, Cat, Sod1, Txnip*) and calcium cycling (*Casq1, Casq2, Atp2a1, Atp2a2, Tmc5*) across three timepoints (WT0, WT30, WT60). Values are centered relative to the mean of 13 housekeeping genes (HK = 0 reference line). A dashed line at 0 reflects this HK baseline, and colors denote timepoint (green = WT0, purple = WT30, pink = WT60). Asterisks indicate statistical significance relative to HK expression at each timepoint (*FDR < 0.05, **FDR < 0.01, ***FDR < 0.001; two-sided t-test with Benjamini-Hochberg correction). *Tmc5* is shown in a separate panel to accommodate its broader expression range. B. Bar plots show the activation-induced gene expression changes with $\Delta\log_2$CPM for each DEG at WT30 and WT60, relative to the average expression change observed in housekeeping genes. Bars are colored based on directionality (purple: upregulated; gray: downregulated). A dashed line at 0 indicates no change relative to housekeeping gene baseline dynamics. Asterisks denote significance based on FDR-adjusted p-values (*FDR < 0.05, **FDR < 0.01, ***FDR < 0.001). Genes are sorted by relative expression change magnitude at each timepoint.

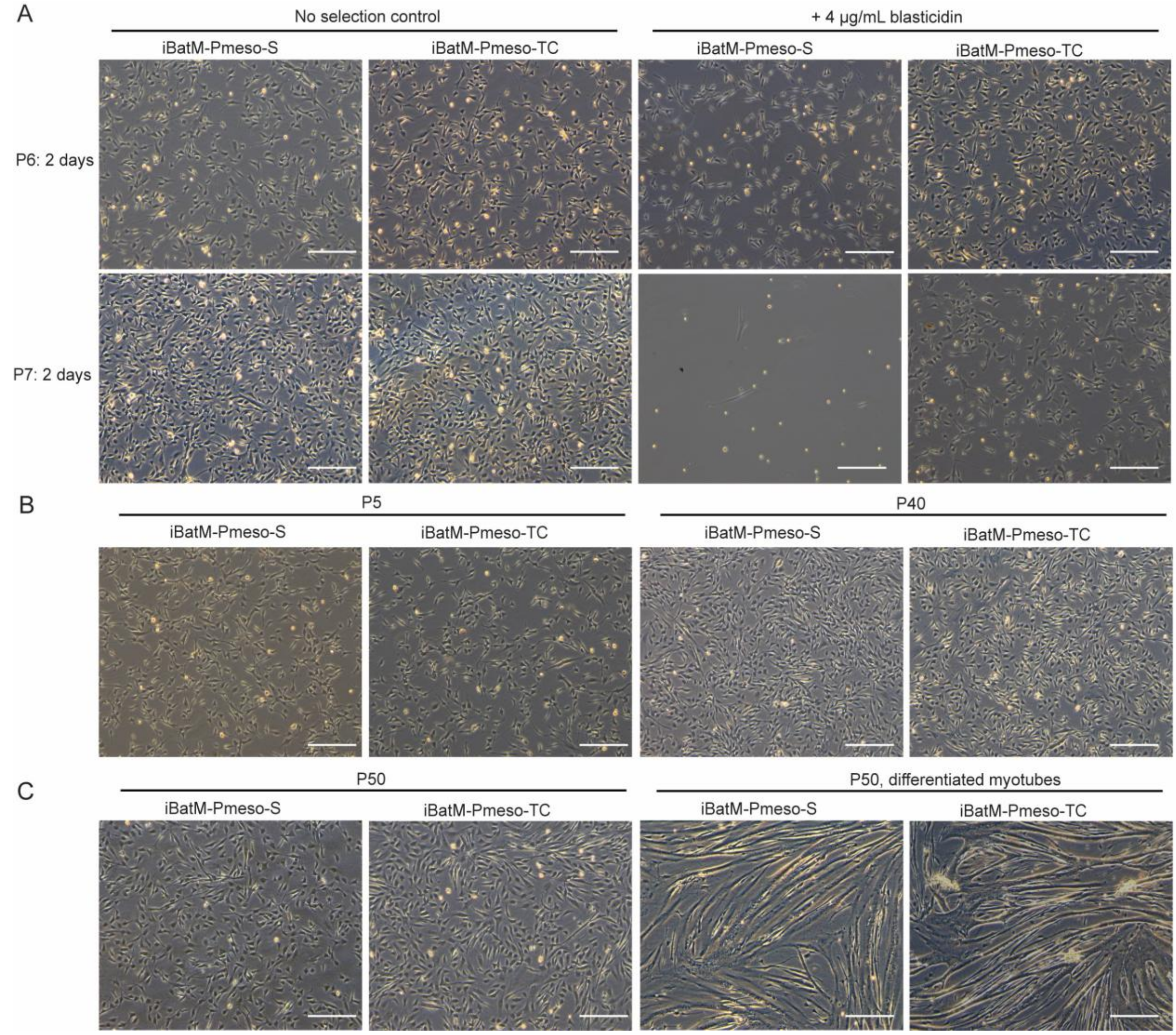

Figure S4. Bat myoblast immortalization. A. Phase-contrast images of primary myblasts from *P. mesoamericanus (*iBatM-Pmeso-S) and hTERT/CDK4 lentivirus transduced myoblasts (iBatM-Pmeso-TC) treated with or without 4 μg/mL blasticidin. Images were taken 2 days post-treatment across multiple passages (P6-7) to assess drug sensitivity and cell viability. B. Phase-contrast images showing the morphology of iBatM-Pmeso-S, and iBatM-Pmeso-TC early (passage 5, left) and late (passage 40, right) stages. C. Phase-contrast images showing the morphology of p50 iBatM-Pmeso-S, p50 iBatM-Pmeso-TC and myotubes differentiated from p50 iBatM-Pmeso-S, p50 iBatM-Pmeso-TC. Scale bar: 100 μm

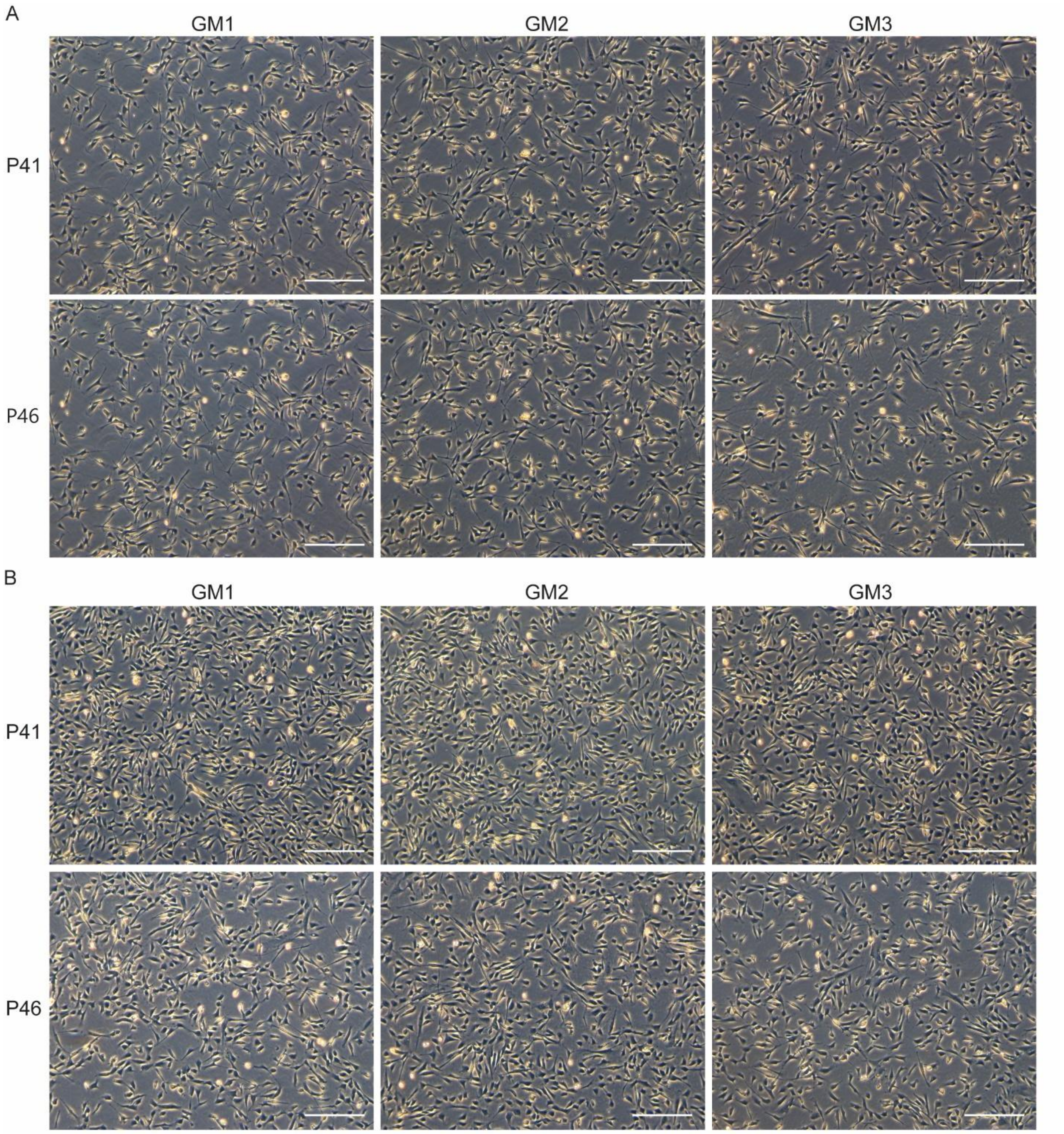

C

GM		GM1	GM2	GM3
Doubling time	iBatM-Pmeso-S	33.35	27.08	20.29
	iBatM-Pmeso-TC	24.37	20.29	23.33

Figure S5. Comparison of Growth Media Effects on immortalized cells. A. Phase-contrast images showing the morphology of self-immortalized myoblasts iBatM-Pmeso-S at passage 41 (P41) and P46 cultured in F10, DMEM/F10, or DMEM-based myoblast growth media. B. Phase-contrast images of hTERT/CDK4-immortalized myoblasts iBatM-Pmeso-TC at different passages cultured under the same media conditions. C. Quantification of doubling time for both iBatM-Pmeso-S and iBatM-Pmeso-TC in F10, 1:1 F10/DMEM, and DMEM, highlighting media-dependent differences in proliferative capacity. Scale bar: 100 μm.

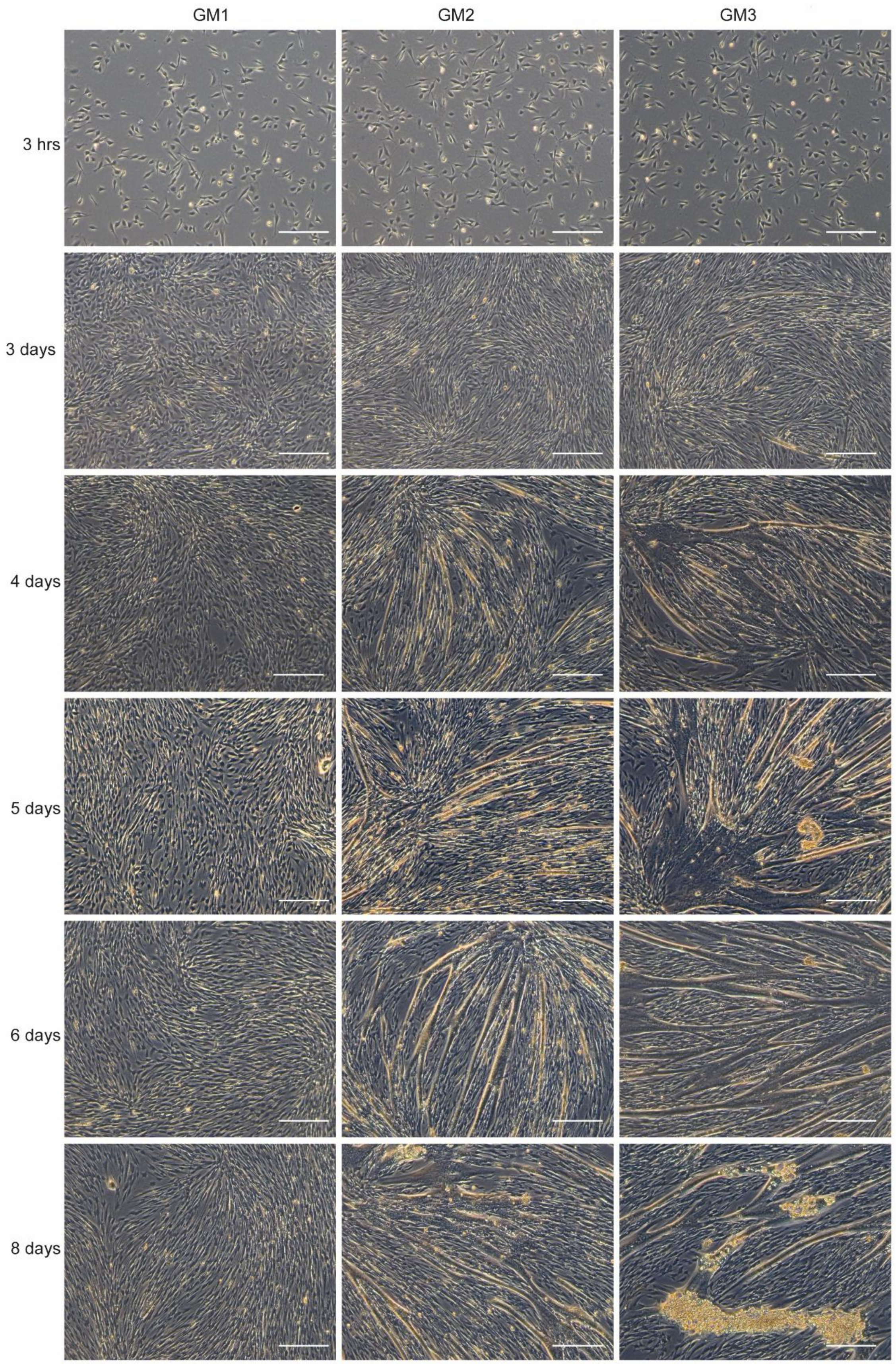

Figure S6. DMEM Media Supports Myotube Formation in iBatM-Pmeso-S. Phase-contrast images showing the morphological progression of iBatM-Pmeso-S cultured in GM1, GM2 and GM3 over an 8-day time course. While all media supported early attachment (3 h) and proliferation (<3 days), only media containing DMEM promoted myotube differentiation by days 6-8. Scale bar: 100 μm.

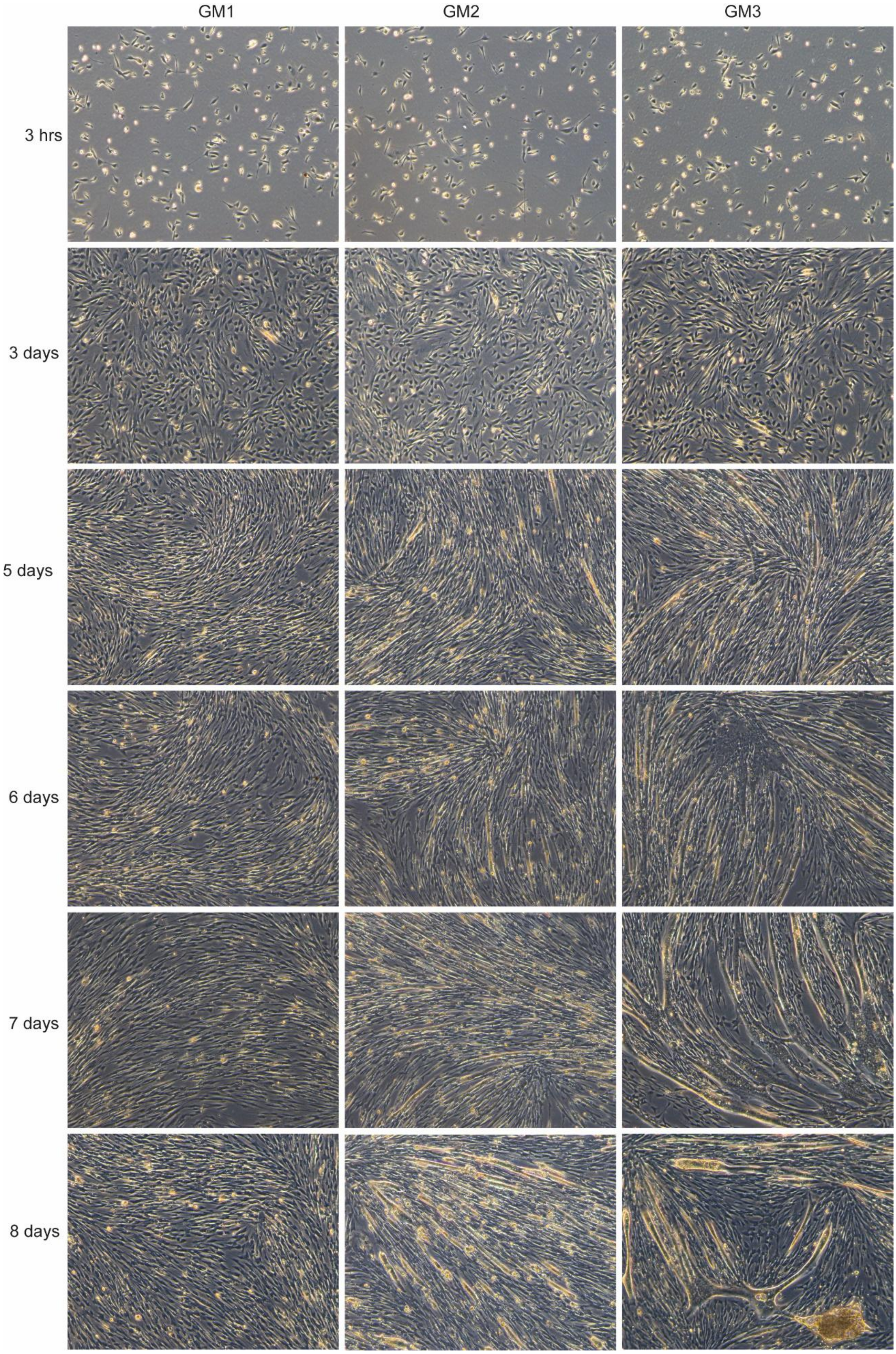

Figure S7. DMEM Media Supports Myotube Formation in iBatM-Pmeso-TC. Phase-contrast images showing the morphological progression of iBatM-Pmeso-TC cultured in GM1, GM2 and GM3 over an 8-day time course. While all media supported early attachment (3 h) and proliferation

(<3 days), only media containing DMEM promoted myotube differentiation by days 6-8. Scale bar: 100 μm.

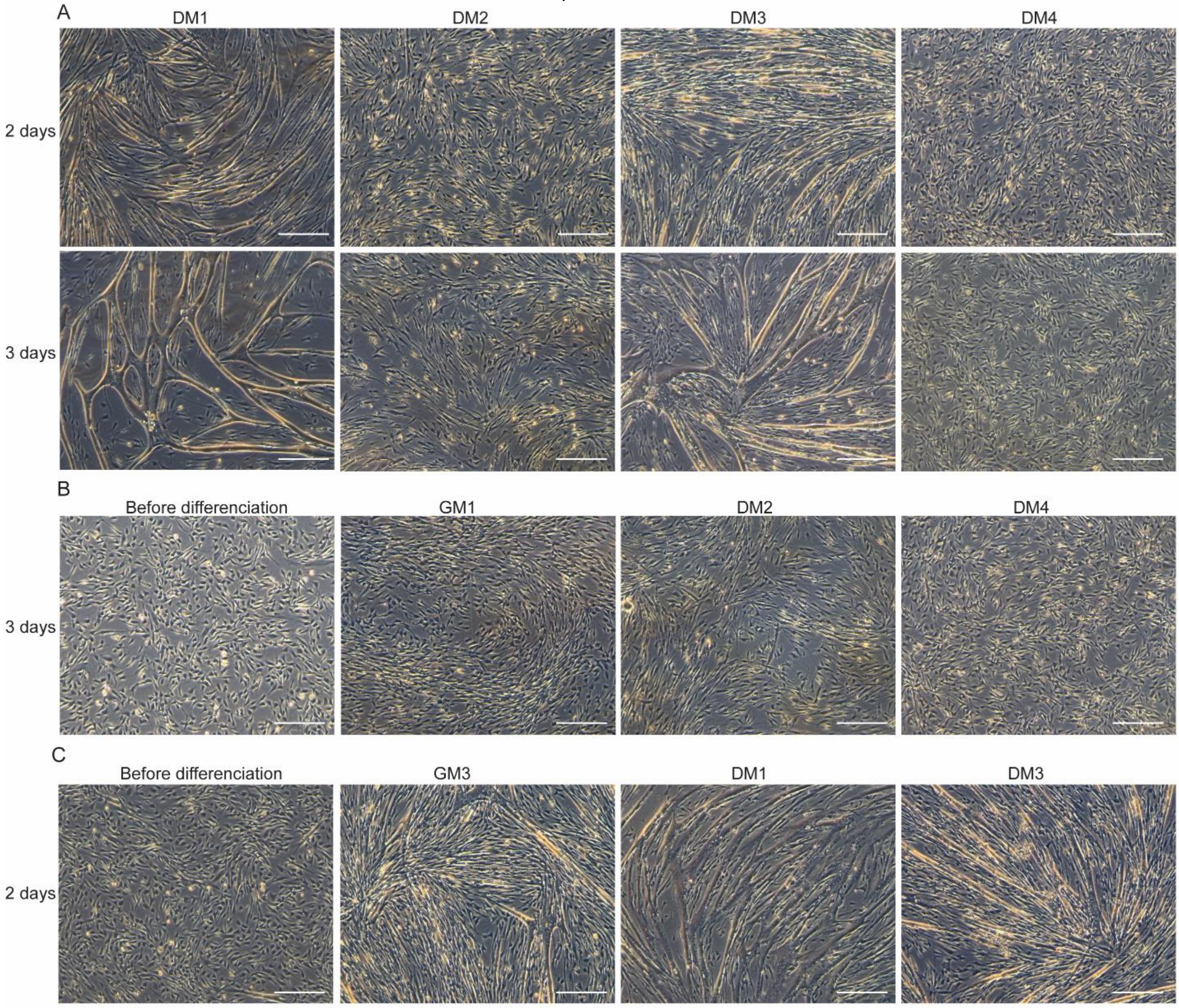

Figure S8. Differentiation of iBatM-Pmeso-S Depend on Media Composition. A. Phase-contrast images of self-immortalized myoblasts (iBatM-Pmeso-S) cultured for 2 and 3 days in four different media formulations (DM1-DM4; Table 2). Morphological differences reflect varying capacities of each media to support myotube formation. B. iBatM-Pmeso-S cultured after 3 days in GM1 versus DM2 and DM4. These conditions failed to support differentiation, as indicated by continued mononuclear morphology and lack of alignment or fusion. C. Comparisons of iBatM-Pmeso-S cultured in GM3, DM1, and DM3 after 2 days. Cell fusion, elongation, and alignment are observed. Scale bar: 100 μm.

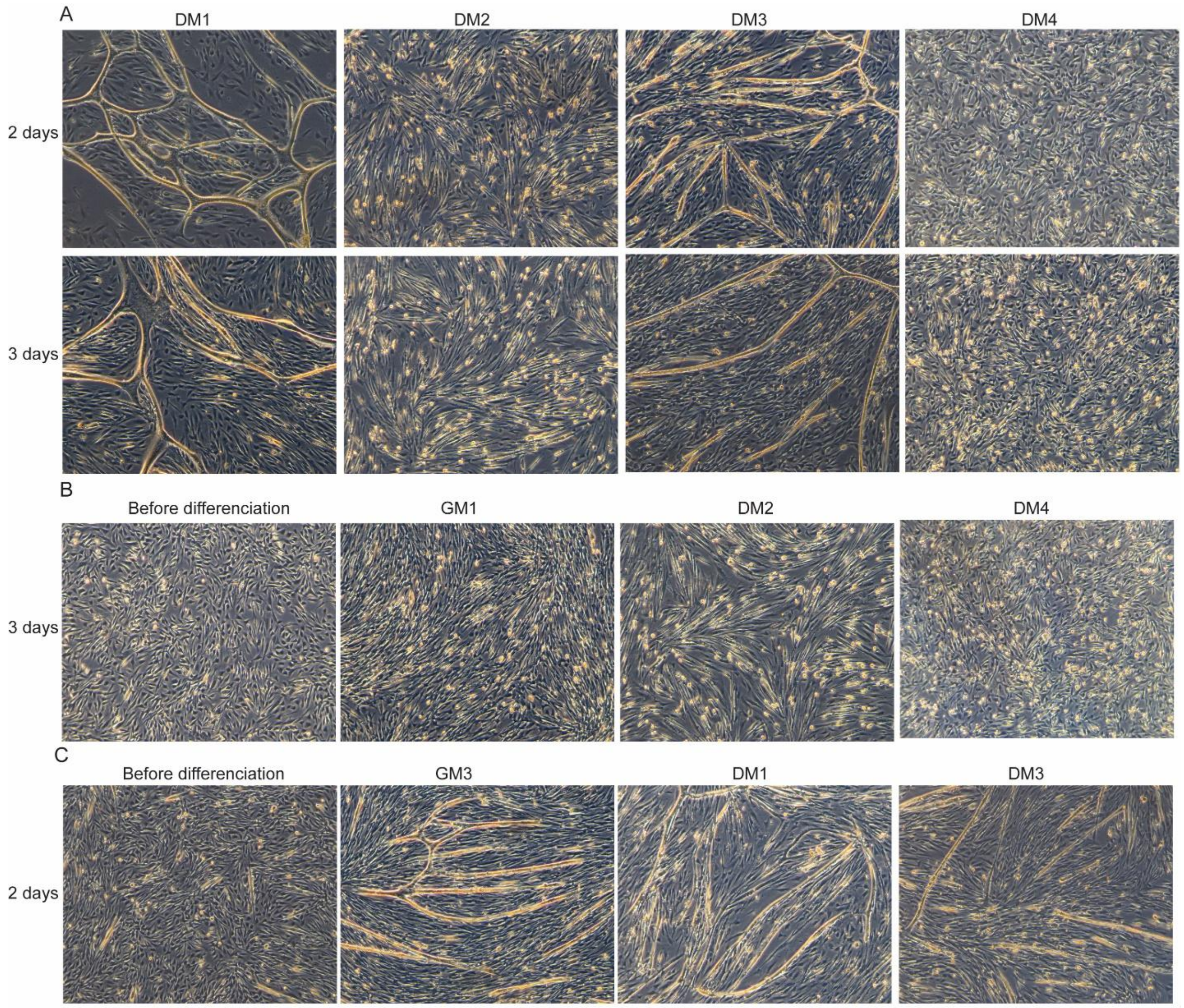

Figure S9. Differentiation of iBatM-Pmeso-TC Depend on Media Composition. A. Phase-contrast images of hTERT/CDK4-immortalized myoblasts (iBatM-Pmeso-TC) cultured for 2 and 3 days in four different media formulations (DM1-DM4; Table 2). B. Cells cultured in GM1, DM2, and DM4 for 3 days. These conditions fail to support clear myotube formation. **C.** 2 days after culturing in DMEM, DM1, and DM3, Cell fusion, elongation, and alignment are observed. Scale bar: 100 μm.

Video S1. Contraction of myotubes differentiated from P2 *Pmeso* primary myoblasts. Video 1 shows spontaneous contraction events over a 17-second recording. Myotubes fused together like a web and the whole field actively contract.

Video S2. Contraction of myotubes differentiated from P43 self-immortalized (iBatM-Pmeso-S) myoblasts. Video 2 shows spontaneous contraction events over a 12-second recording. White arrow indicates the contractile region.

Video S3. Contraction of myotubes differentiated from P43 hTERT/CDK4-immortalized (iBatM-Pmeso-TC) myoblasts. Video 3 shows spontaneous contraction events over a 13-second recording. White arrow indicates the contractile region.
